# Serum Osteocalcin Is Associated with Inflammatory Factors in Metabolic Syndrome: A Population-Based Study in Chinese Males

**DOI:** 10.1155/2015/683739

**Published:** 2015-10-22

**Authors:** Ming Liao, Lirong Huang, Yan Mao, Yonghua Jiang, Ziting Yao, Xinggu Lin, Zheng Lu, Chunlei Wu, Xue Qin, Haiying Zhang, Zengnan Mo

**Affiliations:** ^1^Institute of Urology and Nephrology, First Affiliated Hospital of Guangxi Medical University, Nanning, Guangxi 530021, China; ^2^Center for Genomic and Personalized Medicine, Guangxi Medical University, Nanning, Guangxi 530021, China; ^3^Guangxi Key Laboratory of Genomic and Personalized Medicine, Nanning, Guangxi 530021, China; ^4^Guangxi Collaborative Innovation Center for Genomic and Personalized Medicine, Nanning, Guangxi 530021, China; ^5^Department of Biostatistics and Epidemiology, Perelman School of Medicine, University of Pennsylvania, Philadelphia, PA 19014, USA; ^6^Department of Oncology, Third Affiliated Hospital of Guangxi Medical University, Nanning, Guangxi 530031, China; ^7^Department of Urology, First Affiliated Hospital of Xinxiang Medical College, Xinxiang, Henan 453100, China; ^8^Department of Clinical Laboratory, First Affiliated Hospital of Guangxi Medical University, Nanning, Guangxi 530021, China; ^9^School of Public Health, Guangxi Medical University, Nanning, Guangxi 530021, China; ^10^General Practice School, Guangxi Medical University, Nanning, Guangxi 530021, China

## Abstract

Osteocalcin (OCN) was potentially associated with inflammatory factors, so we explored the metabolic role in this association in general population. Our findings suggest that OCN was positively associated with IgG while inversely associated with C3, both of which were probably mediated by obesity. Moreover, serum OCN was inversely associated with hsCRP in men with impaired fasting glucose, hyperglycemia, or metabolic syndrome, while its association with IgE was significantly observed in men with a normal metabolic profile.

## 1. Introduction

Osteocalcin (OCN) is an osteoblast-derived protein acting as a hormone stimulating insulin sensitivity, insulin secretion, and energy expenditure [1]; however, its effect on immune response and inflammatory cells has not been described before [2–4]. We previously reported that low level of serum total OCN was a potential marker of metabolic syndrome (MetS) that is characteristic of persistent low-grade systemic inflammation [5]. High sensitive C-reaction protein (hsCRP), as a sensitive physiological marker of subclinical systemic inflammation, has been shown to contribute to individual components of MetS [6], but its association with OCN was not clearly studied. In a population-based study assessing the risk of nontraumatic fractures, serum total OCN as a marker of bone turnover showed an inverse relation to hsCRP, without consideration of the metabolic confounders [7]. In studies assessing metabolic factors, the association between serum total OCN and hsCRP appeared to be easily observed in postmenopausal women [8] or young overweight and obese women [9]; however, the other studies showed that the association was independent of sex, in type 2 diabetic patients [10] or old adults (aged ≥ 65 years) [11]. In one study enrolling solely adult males, serum total OCN was inversely associated with CRP in those with risk factors for cardiovascular diseases, but the significant association disappeared after adjusting fasting glucose and free fatty acid [12]. Collectively, the association between hsCRP and OCN was not clearly studied in adult males, especially those with systemic assessment of metabolic factors.

Currently, the putative receptor of OCN (GPRC6A) has been found to be expressed in leukocytes and active in cell mediated immunity, while it is not clear whether OCN or its receptors is active in humoral immunity. The complement system, as a part of the innate immunity, has been found to be involved in the development of metabolic complications [13]. Circulating C3 strongly predicts the cluster of MetS, independent of CRP [14]. Elevated serum C3 confers risk of coronary heart disease, independent of CRP as well [15]. The potential association of OCN with C3 may be distinctive from its association with CRP. Moreover, the adaptive immunity is also involved in the development of MetS. Among the immunoglobulins produced by B cells, IgM, as the first antibody produced during an immune response after an initial antigen counter, is associated with MetS and its individual components in males [16]. IgA concentrations above the reference range are common among male diabetics and associated with diabetic complications [17]. IgG, as the most abundant antibody in the circulation, is also involved in the development of hyperglycemia and obesity [18]. Transfer of IgG from diet-induced obese (DIO) mice rapidly induces insulin resistance and glucose intolerance [19], which could be improved by administration of exogenous OCN [1, 20]. Nevertheless, human studies of association between OCN and inflammatory factors are still lacking. The evidence was so limited to support the hypothesis of potential involvement of OCN in the humoral immunity. So we conducted the present study to explore the association between OCN, hsCRP, complements (C3 and C4), IgM, IgG, IgA, and IgE in adult males. To the best of our knowledge, we are the first to explore the association between OCN and inflammatory factors with systemic assessment of metabolic factors. Our study would provide potential clues of cross talk between bone and immune system and partly support the increasing knowledge in osteoimmunology [21].

## 2. Materials and Methods

### 2.1. Study Population

Our analyses were based on the Fangchenggang Area Male Health and Examination Survey, a population-based cohort study in southern area of Guangxi, China [22]. It was a comprehensive demographic and health survey designed to investigate the effects of environmental and genetic factors and their interaction with the development of age-related chronic diseases. Briefly, a consecutive series of 4,303 men aged 17–88 years participated in the routine physical examination at the Medical Centre in Fangchenggang First People's Hospital from September 2009 to December 2009. A total of 3,593 people completed the data collection with a response rate of 83.5% [23]. There were no significant differences between these people and those who did not complete the interviews. All the participants provided written informed consent, and the present study was approved by the ethics committee in Guangxi Medical University.

Participants with the following conditions were excluded in the present study: (1) currently diagnosed with myocardial infarction, congestive heart failure, stroke, hyperparathyroidism, hyperthyroidism, rheumatoid arthritis, acquired immune deficiency syndrome, and cancer, or in current infection such as influenza, bronchitis, pneumonia, and urethritis; (2) with history of allergic diseases such as asthma and allergic rhinitis, or history of osteoporosis and bone fractures, or taking medications known to influence bone metabolism, such as vitamin D, calcium, calcitonin, bisphosphonate, androgen, estrogen, or corticosteroids; (3) in current treatment with herbal remedies, or with medication including psychotropic drugs, opioids, cimetidine, spironolactone, antibiotics, and nonsteroidal anti-inflammatory drugs; (4) with impaired hepatic function (alanine transaminase >2.0 times upper limit of normal) or with impaired renal function (serum creatinine >178 *μ*mol/L); and (5) having drunk beer, wine, or hard liquor the day before serologic examination. Finally, a total of 2,043 men aged 20–69 years with complete data were available for the present analysis.

### 2.2. Data Collection

A complete physical examination was performed on each subject. The standardized face-to-face questionnaire was used by trained physicians to collect information including smoking status, alcoholic drinking behavior, and health status. Current smokers were defined as smoking at least once a day and lasting for more than 6 months, while former smokers were those who have ceased smoking at least 6 months [24]. Alcoholic drinkers were defined as those who had ever consumed 3 or more drinks (beer, wine, and hard liquor) weekly and done so for 6 consecutive months [24, 25]. The details of anthropometric measurements, including height, weight, (WC), and blood pressure (BP), were carried out by trained physicians. Body mass index (BMI) was calculated as weight in kilograms divided by the square of height in meters. WC was measured at the midpoint between the lower rib margin and the iliac. Both systolic blood pressure (SBP) and diastolic blood pressure (DBP) were measured twice after resting for more than 15 minutes and the average values were obtained.

### 2.3. Serum Assay

The description of the laboratory test has been previously reported in detail [5]. Briefly, about 10 mL overnight venous blood specimens were obtained between 8 AM and 10 AM and transported frozen to the testing center of Department of Clinical Laboratory at the First Affiliated Hospital of Guangxi Medical University in Nanning in two hours. Serums were centrifuged within 15–25 min and stored at −80°C until analysis. High density lipoprotein cholesterol (HDL-c), triglycerides, and glucose were measured enzymatically on a Dimension-RxL Chemistry Analyzer (Dade Behring, Newark, Delaware) in the Department of Clinical Laboratory at the Fangchenggang First People's Hospital. Serum OCN and insulin were measured with electrochemiluminescence immunoassay on COBAS 6000 system E601 (Elecsys module) immunoassay analyzer (Roche Diagnostics, GmbH, Mannheim, Germany) with the same batch of reagents, and the interassay coefficient of variation was 4.5% and 3.2%, respectively. The hsCRP, C3, C4, lgM, lgG, lgA, and IgE levels were detected using the immunoturbidimetric assay on the Hitachi 7600 autoanalyzer (Hitachi Corp, Tokyo, Japan), and the interassay coefficient of variation was 2.20%, 2.16%, 2.51%, 4.67%, 4.97%, 3.57%, and 3.30%, respectively. HOMA-IR of insulin resistance was calculated as follows: ([insulin]*∗*[glucose]/22.5) [26].

### 2.4. Statistical Methods

All statistical analyses were performed using SPSS version 16.0 software (SPSS Inc., Chicago, Illinois). The distribution of continuous variables was examined by Shapiro-Wilks test. Variables were described as proportions for categorical variables and as medians and interquartile ranges for continuous variables with skewed distribution. Because the distribution of OCN levels was skewed, it was transformed by the natural logarithm. In order to maintain the symmetry and comparability of per-unit-effect estimates, the inflammatory factors including hsCRP, complements (C3, C4), and immunoglobulins (IgM, IgG, IgA, and IgE) were all log-transformed in the following analysis. At first, correlations of metabolic factors with OCN and inflammatory factors were examined, and the age-adjusted Pearson's coefficients (*r*) were presented. Then, the associations between OCN and inflammatory factors were evaluated in three linear regression models, and the beta coefficient (*β*) and 95%  CI were present. Briefly, the first model was adjusting for age, then further adjusting for BMI, and further adjusting for other confounders including triglycerides, HDL, fasting glucose, insulin, smoking status, SBP, DBP, and alcoholic drinking behavior. In the subgroup analysis, the associations between OCN and hsCRP, IgE were repeated in the final model adjusting for age, BMI, insulin, smoking status, and alcoholic drinking behavior. As shown in our previous study [22], MetS was defined based on the updated report of National Cholesterol Education Program Adult Treatment Panel III for Asian Americans [27], as having 3 or more of the following components: (1) central obesity: WC ≥ 90 cm, (2) hypertriglyceridemia: triglycerides ≥ 1.7 mmol/L, (3) low HDL: HDL < 1.03 mmol/L, (4) elevated BP: SBP/DBP ≥ 130/85 mmHg or current use of antihypertensive medications, and (5) hyperglycemia: fasting glucose at ≥5.6 mmol/L or being previously diagnosed with type 2 diabetes mellitus or on oral antidiabetic agents or insulin. Participants were defined as having normal glucose tolerance (NGT, fasting glucose < 5.6 mmol/L) or impaired fasting glucose (IFG, fasting glucose = 5.6–7.0 mmol/L) or unknown diabetes (fasting glucose > 7.0 mmol/L) according to American Diabetes Association [28]. Participants were also classified as obese on the basis of the Asian definition of BMI ≥ 27.5 kg/m^2^ [29]. Statistical tests were two-sided, and a *P* < 0.05 was considered statistically significant.

## 3. Results

The baseline demographic characteristic of the 2,043 participants was shown in Table 1. Overall, the median age of the sample was 36 years (interquartile range: 29–45), and median serum OCN was 22.9 ng/mL (interquartile range: 18.8–28.6). OCN was significantly correlated with age (*r* = −0.341, *P* < 0.001).  Table 2 provided the age-adjusted Spearman partial correlation coefficients between OCN, hsCRP, complements (C3, C4), IgM, IgG, IgA, IgE, and metabolic factors. The OCN was inversely correlated with hsCRP (*r* = −0.144, *P* < 0.001), C3 (*r* = −0.123, *P* < 0.001), C4 (*r* = −0.073, *P* = 0.001), and IgE (*r* = −0.057, *P* = 0.034), while being positively correlated with IgG (*r* = 0.048, *P* = 0.034). In terms of metabolic factors, OCN was inversely correlated with BMI, WC, SBP, DBP, glucose, triglyceride, HOMA-IR, and insulin, while these metabolic factors were positively correlated with hsCRP, C3, and C4. Moreover, IgG was inversely correlated with BMI, and IgM was inversely correlated with WC, while IgE was inversely correlated with BMI and WC.

The associations between OCN and hsCRP, immunoglobulins, and complements were analyzed in the linear regression models as shown in Table 3. OCn was significantly associated with age (*β* = −0.010, 95%  CI = −0.011, −0.009, and *P* < 0.001). In age-adjusted model, OCN was inversely associated with hsCRP (*β* = −0.034, 95%  CI = −0.044 to −0.024, and *P* < 0.001), C3 (*β* = −0.171, 95%  CI = −0.231 to −0.111, and *P* < 0.001), C4 (*β* = −0.079, 95%  CI = −0.126 to −0.033, and *P* < 0.001), and IgE (*β* = −0.013, 95%  CI = −0.022, −0.003, and *P* = 0.005). In the age-BMI-adjusted model, the inverse association between OCN and hsCRP (*β* = −0.014, 95%  CI = −0.024, −0.003, and *P* < 0.001) and IgE (*β* = −0.016, 95%  CI = −0.025, −0.007, and *P* = 0.001) remained statistically significant, and these associations were not attenuated in the model with further adjusting for triglycerides, HDL, glucose, insulin, SBP, DBP, smoking status, and alcoholic drinking behavior.

The associations between OCN and hsCRP and IgE were further explored in the subgroups as shown in Table 4. The inverse association between OCN and hsCRP was obviously observed in men with MetS (*β* = −0.046, 95%  CI = −0.088 to −0.004, and *P* = 0.032) and men with hyperglycemia (*β* = −0.039, 95%  CI = −0.059 to −0.018, and *P* < 0.001) or IFG (*β* = −0.035, 95%  CI = −0.057 to −0.0148, and *P* = 0.001) or BMI ≥ 27.5 kg/m^2^ (*β* = −0.039, 95%  CI = −0.075 to −0.002, and *P* = 0.039). Meanwhile, the inverse association between OCN and IgE was only observed in men without MetS (*β* = −0.017, 95%  CI = −0.027 to −0.008, and *P* < 0.001) and men with NGT (*β* = −0.019, 95%  CI = −0.029 to −0.008, and *P* < 0.001) or BMI < 27.5 kg/m^2^ (*β* = −0.015, 95%  CI = −0.025 to −0.006, and *P* = 0.020) or men without each individual component of MetS factors. Moreover, the inverse association of OCN with both hsCRP and IgE was statistically significant in men with only one component of MetS factors.

## 4. Discussions

In this large cross-sectional study of Chinese male population with systemic assessment of metabolic factors, serum OCN was inversely associated with hsCRP, especially in men with IFG, hyperglycemia, or MetS. The potential association between OCN and C3 may be mediated by obesity. OCN was not associated with IgM and IgA but inversely associated with IgE in men with a normal metabolic profile.

Although the inverse association between OCN and hsCRP has been reported, we are the first to show this association in adult males with prediabetes or MetS. As shown in another Chinese study enrolling adult males with risk factors for cardiovascular diseases, the age-adjusted inverse association between OCN and CRP was observed in men with NGT and normal BMI but was not statistically significant after further adjusting for visceral fat area [12]. In the present study, the inverse association between OCN and hsCRP was statistically significant in men with IFG, hyperglycemia, or MetS, even after adjusting metabolic confounders including BMI or WC. In the European study of nondiabetic overweight and obese adults, OCN was inversely associated with hsCRP after adjusting for age, physical activity, and fat mass, but this significant association was only observed in the female subjects [9], consistent with the results from postmenopausal women [8]. On the one hand, the sample size of male subjects (*N* = 116) in this study was probably too small to detect the genuine association between OCN and CRP in males. On the other hand, gender difference may exist in the potential association between OCN and CRP, as postmenopausal women had a lower level of CON and a higher level of CRP than middle-aged and elderly men [8], while young women had a higher level of OCN and a lower level of CRP than young men [9]. Similarly, ethnic/racial difference may exist as well [11, 30]. As shown in a multiethnic study of school children, OCN was significantly higher in East Asian Americans than the other ethnic/racial groups [30]. Although the gender or ethnic/racial difference has not been confirmed, the inverse association between OCN and hsCRP in IFG or hyperglycemia may shed new light on the anti-inflammatory role of OCN in maintaining glucose homeostasis.

To the best of our knowledge, we are the first to explore the association between OCN and complements C3, C4. Consistent with previous studies, we found that C3 was positively associated with metabolic factors including fasting triglycerides, WC, HOMA-IR, and BMI [14]. So was the association between OCN and MetS, as we previously showed that low level of serum OCN was a risk factor for MetS [5]. Thus, it was not surprising to find out that OCN was inversely associated with C3 in the present study. However, this age-adjusted association was not statistically significant after further adjusting for BMI or WC. Because adipose tissue can produce both C3 and OCN, this association between OCN and C3 may be affected by BMI or WC, two important proxies of obesity. Considering the contribution of complement in the low-grade inflammation associated with obesity, OCN may be involved in the pathways of complement activation during metabolic inflammation [31]. Although we cannot rule out the possibility that OCN was associated with C3 independent of metabolic factors, OCN as one of the biomarkers secreted by osteoblast cells may be related to the inflammatory process involving complements [32].

Immunoglobulins were potentially associated with MetS, but their association with OCN has not been studied before. As shown in the previous study, serum IgM concentration was positively associated with MetS in an adult population [16]. In the mice model, the hyperglycemia alone can delay production of IgM [33]. Although OCN may play a beneficial role in the development of hyperglycemia, its effect on the production of IgM has not been identified. Our findings suggested OCN was not associated with IgM, but further researches are needed to verify it. In DIO mice, not only reduced production of IgM but also increased IgG secretion were observed [19]. The transfer of purified IgG from DIO mice but not from lean mice induces an Fc-dependent worsening of insulin resistance, suggesting the linkage between IgG and obesity-related insulin resistance [19]. In the present study, the association between IgG and OCN was initially observed, but this age-adjusted association was not statistically significant after further adjusting for BMI or WC. Adipose tissue seems to play a crucial role in the association between IgG and OCN [18]. Because there are Fc receptors in human adipose tissue [34], the IgG may have direct effects on adipocyte function and leads to OCN production [35]. Because IgA exists primarily as a monomer in serum, its exposed Fc domains may relate to the Fc-dependent function on adipocytes as well. But in the present study, IgA was not associated with OCN, probably due to the J chain in the polymeric forms of IgA, which bonds with the Fc domains of IgA and disturbs the Fc-dependent function on adipocytes. In the present study, only IgE was significantly associated with OCN, and this association was obviously observed in healthy adults without each component of MetS. MetS is characterized by an aggregate of interrelated metabolic factors consisting of insulin resistance, visceral obesity, and atherogenic dyslipidemia. The significant association between OCN and IgE was still observed in men with only one component of MetS. These findings may suggest the important role of OCN and IgE in the development of MetS. As shown in the rat model, the cholesterol accumulation within the macrophages of atherosclerotic lesion was IgE-dependent [36]. Although the effect of OCN on the IgE-activated mast cells has not been identified, effective mast cell inhibitor medications used in preformed obesity and diabetes in experimental models offer hope to patients with these common chronic inflammatory diseases [37].

Our study was strengthened by the large sample size of the cohort population with a wide range of age. We also controlled the potential confounding of traditional metabolic factors in data analyses. However, we recognized several limitations in the present study. Firstly, given the nature of cross-sectional study, we could not establish causal relationships between OCN and the inflammatory factors. Secondly, we only measured hsCRP, complements, and immunoglobulins as inflammatory markers but did not evaluate other markers such as tumor necrosis factor *α* and IL-6, which were potentially associated with obesity or type 2 diabetes [38]. Particularly, assessment of testosterone would be of high value in systemic inflammation-based vasculopathy and metabolic syndrome [39]. Last but not least, the study population consisted solely of men, and only total OCN was measured but not its uncarboxylated or carboxylated form. Considering the potential differences between total OCN and uncarboxylated OCN and the differences between males and females, further well-designed study is needed to explore the role of OCN in the inflammation related to metabolic disorders. Despite these limitations, this is the first cross-sectional study to investigate the relation between OCN and inflammatory factors in general population.

In conclusion, our study tried to clarify the role of metabolic factors in the association between OCN and the inflammatory factors. We find out that serum OCN was not associated with IgM and IgA, while its potential association with IgG and C3 was probably mediated by obesity. Moreover, serum OCN was inversely associated with hsCRP in men with IFG, hyperglycemia, or MetS, while being inversely associated with IgE in men with a normal metabolic profile. Our findings may support the possibility of using OCN in the prediction or treatment for metabolic disorders.

## Figures and Tables

**Table 1:** Baseline demographic characteristics (*n* = 2,043).

Variable	Median (25–75th percentiles) or *n* (%)
Age (yr)	36 (29–45)
<30	554 (27.1)
30–40	773 (37.8)
>40	716 (35.0)
Smoking status	
Current smoker	1028 (50.3)
Former smoker	84 (4.1)
Never smoked	931 (45.6)
Alcoholic drinker	
Yes	1750 (85.7)
No	29 (14.3)
Physical examination	
Body mass index (kg/m^2^)	23.1 (20.8–25.5)
Waist circumference (cm)	80.5 (73.5–87.5)
Systolic blood pressure (mmHg)	120 (110–126)
Diastolic blood pressure (mmHg)	80 (70–80)
Osteocalcin (ng/mL)	22.9 (18.8–28.6)
Inflammatory factors	
High sensitivity C-reactive protein (mg/L)	0.55 (0.25–1.28)
Complement C3 (IU/mL)	1.11 (0.97–1.26)
Complement C4 (IU/mL)	0.32 (0.27–0.38)
IgM (IU/mL)	1.23 (0.9–1.7)
IgG (IU/mL)	13.2 (11.5–14.9)
IgA (IU/mL)	2.36 (1.79–2.97)
IgE (IU/mL)	126.6 (51.8–317.0)
Metabolic factors	
HDL (mmol/L)	1.35 (1.18–1.57)
Triglycerides (mmol/L)	1.13 (0.78–1.76)
Fasting glucose (mmol/L)	5.2 (4.9–5.6)
Insulin (mlU/L)	6.5 (4.42–9.76)
HOMA	0.83 (0.57–1.27)

**Table 2:** Age-adjusted Spearman partial correlations between log (osteocalcin), log (hsCRP), log (inflammatory factors), and log (metabolic factors).

	Osteocalcin	hsCRP	C3	C4	IgM	IgG	IgA	IgE
Osteocalcin		−0.144^c^	−0.123^c^	−0.073^b^	0.007	0.048^a^	0.014	−0.057^a^
BMI	−0.292^c^	0.312^c^	0.402^c^	0.223^c^	−0.037	−0.050^a^	−0.022	−0.047^a^
WC	−0.267^c^	0.320^c^	0.407^c^	0.227^c^	−0.061^b^	−0.092	−0.011	−0.056^b^
SBP	−0.059^b^	0.109^c^	0.127^c^	0.083^c^	−0.035	−0.023	0.042	0.007
DBP	−0.113^c^	0.129^c^	0.169^c^	0.085^c^	−0.056^a^	−0.014	0.056^a^	−0.013
Glucose	−0.107^c^	0.107^c^	0.121^c^	0.062^b^	−0.001	0.001	0.057^a^	0.028
Triglyceride	−0.199^c^	0.223^c^	0.279^c^	0.110^c^	−0.006	−0.130	−0.039	−0.022
HDL	0.079^c^	−0.180^c^	−0.246^c^	−0.152^c^	−0.014	−0.016	−0.036	0.052^a^
HOMA	−0.145^c^	0.213^c^	0.278^c^	0.138^c^	−0.011	−0.025	0.061^b^	−0.007
Insulin	−0.140^c^	0.247^c^	0.358^c^	0.152^c^	−0.057^a^	−0.029	0.045^a^	−0.009

WC: waist circumference, SDP: systolic blood pressure, and DBP: diastolic blood pressure.

^a^ *P* < 0.05, ^b^ *P* < 0.01, and ^c^ *P* < 0.001, respectively (all two-tailed).

**Table 3:** Linear regression coefficients and 95% CI for log (osteocalcin) as the dependent variable and log (hsCRP) and log (inflammatory factors) as independent variables.

Inflammatory factors	Model 1		Model 2		Model 3	
	*β*	95% CI	*β*	95% CI	*β*	95% CI
hsCRP	−0.034^c^	(−0.044, −0.024)	−0.014^a^	(−0.024, −0.003)	−0.012^a^	(−0.023, −0.002)
C3	−0.171^c^	(−0.231, −0.111)	−0.010	(−0.013, −0.010)	−0.017	(−0.082, 0.048)
C4	−0.079^b^	(−0.126, −0.033)	−0.009	(−0.055, 0.037)	−0.014	(−0.061, 0.032)
IgM	0.004	(−0.022, 0.031)	−0.002	(−0.028, 0.023)	−0.006	(−0.031, 0.020)
IgG	0.068^a^	(0.006, 0.129)	0.047	(−0.013, −0.010)	0.026	(−0.035, 0.087)
IgA	0.012	(−0.024, 0.047)	0.006	(−0.027, −0.040)	0.000	(−0.034, 0.035)
IgE	−0.013^b^	(−0.022, −0.003)	−0.016^b^	(−0.025, −0.007)	−0.015^a^	(−0.024, −0.006)

Model 1 was adjusted for age; model 2 was further adjusted for BMI; model 3 was adjusted for model 2 plus triglycerides, HDL, fasting glucose, insulin, smoking status, systolic blood pressure, diastolic blood pressure, and alcoholic drinking behavior.

^a^ *P* < 0.05, ^b^ *P* < 0.01, and ^c^ *P* < 0.001, respectively (all two-tailed).

**Table 4:** Multivariant-adjusted association between log (osteocalcin) and log (hsCRP) and log (IgE) in subgroups.

	*n*	OCN	hsCRP			IgE		
		Median	*β*	(95% CI)	*P*	*β*	(95% CI)	*P*
Metabolic syndrome								
No (<3 components)	1851	23.42 (19.15–29.18)	−0.009	(−0.020, 0.002)	0.093	−0.02	(−0.027, −0.008)	<0.001
Yes (≧3 components)	192	19.63 (16.75–23.67)	−0.046	(−0.088, −0.004)	0.032	0.002	(−0.029, 0.033)	0.893
Hypertriglyceridemia								
No (TG <1.7 mmol/L)	1481	23.81 (19.47–29.46)	−0.01	(−0.021, 0.002)	0.096	−0.02	(−0.030, −0.009)	<0.001
Yes (TG ≧1.7 mmol/L)	562	21.03 (17.53–26.07)	−0.021	(−0.046, 0.006)	0.126	−0	(−0.021, 0.015)	0.747
Hyperglycemia								
No (GLU <5.6 mmol/L)	1492	23.71 (19.525–29.41)	−0.003	(−0.015, 0.009)	0.608	−0.02	(−0.030, −0.009)	<0.001
Yes (GLU ≧5.6 mmol/L)	551	21.03 (17.57–26.34)	−0.039	(−0.059, −0.018)	<0.001	−0.01	(−0.025, 0.011)	0.454
Elevated BP								
No (<130/85 mmHg)	1838	23.37 (19–29.09)	−0.013	(−0.024, −0.002)	0.022	−0.02	(−0.025, −0.006)	0.001
Yes (≧130/85 mmHg)	205	20.81 (17.39–25.68)	−0.006	(−0.040, 0.029)	0.752	−0.02	(−0.046, 0.008)	0.167
Low HDL-c								
No (≧1.03 mmol/L)	1894	23.22 (18.84–29.04)	−0.011	(−0.021, 0.001)	0.057	−0.02	(−0.026, −0.007)	<0.001
Yes (<1.03 mmol/L)	149	20.92 (18.47–24.05)	−0.014	(−0.055, 0.023)	0.488	−0	(−0.043, 0.039)	0.928
Central obesity								
No (WC <90 cm)	1687	23.64 (19.45–29.39)	−0.01	(−0.021, 0.001)	0.075	−0.02	(−0.026, −0.006)	0.002
Yes (WC ≧90 cm)	356	19.86 (16.84–24.88)	−0.022	(−0.052, 0.008)	0.158	−0.01	(−0.032, 0.012)	0.365
Obesity according to BMI								
<27.5 kg/m^2^	1821	23.38 (19.07–29.14)	−0.009	(−0.020, −0.002)	0.096	−0.02	(−0.025, −0.006)	0.002
≧27.5 kg/m^2^	222	20.69 (16.6–24.37)	−0.039	(−0.075, −0.002)	0.039	−0.01	(−0.038, 0.015)	0.394
Premetabolic syndrome								
None	967	25.17 (20.67–31.27)	<0.001	(−0.015, 0.014)	0.945	−0.02	(−0.031, −0.005)	0.005
1 component	574	22.21 (18.07–27.57)	−0.031	(−0.051, −0.012)	0.002	−0.02	(−0.037, −0.003)	0.02
≧2 components	310	20.61 (17.38–24.91)	0.002	(−0.030, 0.034)	0.892	−0.01	(−0.030, 0.018)	0.623
Prediabetes								
NGT (GLU <5.6 mmol/L)	1796	23.71 (19.53–29.41)	−0.004	(−0.016, 0.008)	0.529	−0.02	(−0.029, −0.008)	<0.001
IFG (GLU = 5.6–7.0 mmol/L)	558	21.43 (17.94–26.86)	−0.035	(−0.057, −0.014)	0.001	0.002	(−0.017, 0.021)	0.84
Unknown diabetes (GLU >7.0 mmol/L)	70	18.42 (15.47–22.7)	−0.036	(−0.103, 0.031)	0.285	−0.05	(−0.103, 0.008)	0.093

Adjusted for age, BMI, insulin, smoking status, and alcoholic drinking behavior.

OCN: osteocalcin, hsCRP: high sensitive C-reaction protein, TG: triglycerides, GLU: fasting glucose, BP: blood pressure, HDL-c: high density lipoprotein cholesterol, WC: waist circumference, BMI: body mass index, NGT: normal glucose tolerance, and IFG: impaired fasting glucose.
